# Measuring Dietary Restraint Status: Comparisons between the Dietary Intent Scale and the Restraint Scale

**DOI:** 10.3389/fnut.2015.00008

**Published:** 2015-04-01

**Authors:** Jessica A. Boyce, David H. Gleaves, Roeline G. Kuijer

**Affiliations:** ^1^Department of Psychology, University of Canterbury, Christchurch, New Zealand; ^2^School of Psychology, Social Work, and Social Policy, University of South Australia, Adelaide, SA, Australia

**Keywords:** Dietary Intent Scale, Restraint Scale, restrained eaters, dietary restraint status, body image, food intake, weight change, college women

## Abstract

The measurement of young women’s self-reported dietary restraint status is complex. Compared to Herman and Polivy’s commonly utilized Restraint Scale (RS), Stice’s Dietary Intent Scale (DIS) is less understood. Because the DIS is becoming a popular research tool, it is important to understand how this scale compares to more traditional measures of restraint. We conducted two correlational studies (Study 1 *N* = 110; Study 2 *N* = 216) to ascertain the similarities and the differences between the DIS and – as a comparison measure – the well-researched RS. We explored how the two scales were related to several body image variables (e.g., thin-ideal internalization); with a range of self-regulatory variables (e.g., dispositional self-control); with observed food intake during a taste test; and with 18-month weight change (Study 2 only). Participants were female University students and were not selected for dieting or disordered eating. Unlike RS scores, DIS scores were not significantly correlated with the majority of variables tapping into unsuccessful self-regulation. However, our data also highlighted similarities between the two restraint scales (e.g., association with 18-month weight-*loss*) and demonstrated that not only were participants’ DIS scores un-related to unsuccessful self-regulatory variables, neither were they related to the variables tapping into successful self-regulation.

## Introduction

Dieting is defined as “the intentional and sustained restriction of caloric intake for the purpose of reducing body weight or changing body shape, resulting in a significant negative energy balance” (p. 2582) (1). In comparison, restrained eating is broadly defined as the *intention* to restrict food intake to achieve or maintain a more desirable body weight (2).

Researchers usually employ one of three self-report scales to identify restrained eaters within the general population: Herman and Polivy’s Restraint Scale (RS) (3), the restraint subscale from van Strien, Frijters, Bergers, and DeFares’ Dutch Eating Behavior Questionnaire (DEBQ-R) (4), or the restraint subscale from Stunkard and Messick’s Three Factor Eating Questionnaire (TFEQ-R/eating inventory EI) (5). However, measuring dietary intentions *and* behaviors via self-report is challenging and has a complex history. Caution needs to be taken when research based on “restrained eaters” is applied to “real-world” dieters.

The dietary restraint literature is plagued by two problems. First, researchers commonly use participants’ restraint scale scores to connect dietary restriction with negative psychological and physical restraint-related effects (e.g., guilt, overeating, weight gain) (6). However, in recent years, researchers have been criticized for basing such connections on self-report dietary restraint scales that are measuring body image and eating *attitudes*, rather than *behavioral* dietary restriction (2). Second, researchers tend to generalize from one measure to another measure, without acknowledging the differences between such restraint scales. Nonetheless, it has now been well established that the previously referred to restraint-related effects (e.g., overeating) are scale specific (7). Therefore, before claiming that dieting causes overeating or weight-gain, one needs to understand the intricacies of their chosen restraint scale and the commonalities between scales.

For researchers to best advance the literature and to make informed decisions about the most appropriate scale for their research question, it must be clear what constructs each restraint scale measures. Because a multitude of research has already compared the three aforementioned scales (2), we endeavored to gather more information about Stice’s newer Dietary Intent Scale (DIS) (8, 9). We employed the most commonly utilized restraint scale, Herman and Polivy’s RS (3), as a comparison measure.

As noted by Herman, Polivy, and colleagues “The RS was initially proposed (10) as a simple and relatively straightforward self-report device for identifying chronic dieters” (p. 19) (11). As might be expected, women who score highly on the RS tend to be concerned with their body image (12, 13). Perhaps unexpectedly, RS scores predict weight gain, rather than behavioral dietary restriction (14, 15). We use the term *unexpectedly* because the title of the scale suggests that the RS items would measure dietary restriction. However, Herman and Polivy admit that the initial labeling of the “restraint” scale has led to confusion in the literature (11). They argue that the RS was actually intended to measure a range of on/off chronic dieting behaviors, such as restraint and restraint-related effects (e.g., guilt and overeating induced by the burden of constant cognitive restraint – cf. restraint theory) (16). Moreover, soon after the development of the RS, the scale was found to be multidimensional and consisting of two subscales – concern for dieting (RS-CD, “do you eat sensibly in front of others and splurge alone”) and weight fluctuations (RS-WF, “in a typical week how much does your weight fluctuate”) (17, 18). In comparison, other similarly named restraint scales (e.g., DEBQ-R and TFEQ-R) were designed to exclusively measure more successful dietary restriction, which is not commonly interrupted by disinhibition and guilt (11). However, although the authors of the RS may not have designed their scale to measure dietary *restriction* similar to dieting, it has been employed in this way for over two decades among the general population of women (i.e., non-clinical samples) (19–21).

The DIS items (e.g., “I count calories to try to prevent weight gain”) were designed to measure weight-loss and maintenance behaviors, and the intention to diet over the previous 6 months (8, 9). Similar to the RS, DIS scores correlate highly with measures of body shame and anxiety (22). However, because of its limited use, it is less clear how participants’ DIS scores relate to behavioral dietary restriction, self-regulation, and weight-change. Previous researchers tested whether or not scores on the DIS, the RS, and the DEBQ-R correlated significantly with unobtrusively observed eating (8). Unlike participants’ RS scores, their DIS and DEBQ-R scores correlated negatively with fat-gram intake in a fast food restaurant. Participants’ DIS scores also correlated negatively with total caloric intake. Although these results imply that the DIS measures successful dietary restriction, it is noteworthy that these negative correlations have not been replicated (23, 24).

Because of the increasing number of researchers using Stice’s DIS in their research (22, 25), our overriding objective was to pinpoint the attitudes, behaviors, and weight-trajectories that the DIS measures in the general population of women. As mentioned, the RS was chosen as a comparison scale because it is the most frequently used and well-researched dietary restraint scale. Future research will benefit from understanding how past and current restraint scales “hang together.”

In an effort to capture the various attitudes and behaviors associated with dietary restraint status, we had female participants complete both restraint scales (RS and DIS) and a taste test, and also a series of body image, individual-difference, and self-regulatory scales. Although there are similarities between our current studies and the previously referenced studies of the DIS (8, 23, 24), we improved past methodology by incorporating a wider range of variables and (in Study 2 only) measuring 18-month weight change. To our best knowledge, no study has tested the ability of the DIS to predict body-weight change in a non-clinical sample.

## Study 1 Introduction

In both Studies 1 and 2, we measured BMI, weight-dissatisfaction, social comparison tendency, and food intake. In this first study, we also measured body-image investment and eating expectancies. Previous research has demonstrated that women are dissatisfied with their bodies and invested in their body image because they are prone to compare and evaluate themselves against other women (26). We measured both social comparison and body-image investment because they are connected to problematic body-image and eating behavior (27, 28). We also assessed the extent to which participants expect eating to act as a reinforcer (i.e., eating expectancies) – a characteristic that correlates with measures of eating and overeating (29–31).

## Study 1 Method

### Participants

One hundred and twenty-two students completed the study. However, because obese participants (BMI ≥ 30) score highly on the RS-WF scale, their high scores on the total RS scale are more reflective of their high body mass, rather than a high level of dietary concern or restraint (32). Consequently, the RS becomes less internally consistent when it is analyzed with the data gathered from obese participants (2). Therefore, we excluded data from seven obese participants for data analysis. In addition, because it is likely that the inclusion of underweight participants would skew the results, five underweight participants (BMI < 18.5) were also excluded from data analyses. This exclusion has the added benefit of being able to generalize the results to the majority of the population – normal weight and overweight women. The final sample consisted of 110 participants with a mean age of 22.57 years (SD = 5.71, range 18–54) and mean BMI of 23.12 (SD = 3.11, range 18.67–29.75). Eighty participants were classified as normal weight (18.5 ≥ BMI < 25) and 30 as overweight (BMI ≥ 25). Sixty-nine percent of the sample identified themselves as New Zealand European, 7% identified as Chinese, and 5% identified as New Zealand European and New Zealand Māori. The remaining 19% of the sample self-identified as *other* ethnicities (e.g., North American or European). Participants were not screened or selected for weight-loss or dieting intentions.

### Measures and procedure

This study received approval from the campus Human Ethics Committee. Female participants were recruited via the university’s psychology participant pool and via email advertisements sent around other university departments. Participants were offered $10 or course credit to complete the study. Data were collected alongside of another study that was advertised as an investigation into hunger and memory (33). The majority of data were obtained via online self-report questionnaires: restraint status, weight dissatisfaction, social comparison orientation, body image investment, and endorsement of eating expectancies. Two weeks later, as part of a supposedly separate study, participants also completed a 10-min taste test and – prior to debriefing for both studies – the investigator weighed participants with a digital scale and recorded their height to calculate a body mass index (BMI = kg/m^2^). At the conclusion of the study, participants were debriefed and provided with contact information for the Ministry of Health’s Healthline service (free health advice from trained nurses) and for student health and counseling on campus should they want to discuss any weight or body image concerns.

#### Dietary restraint status

Participants completed the nine-item DIS (9). They responded to this measure on a five-point scale, ranging from (1) *never* to (5) *always*. In two separate studies, Stice and his colleagues (8, 9) found scores on this scale to be internally consistent (Cronbach’s α = 0.93) and temporally reliable (1-month test/retest = 0.92). Cronbach’s α for the present study was 0.87 and total scores ranged from 1.00 to 4.89. Participants also completed the 10-item RS (3). Scores on this scale have generally been found to be internally consistent (Cronbach’s α > 0.80) and temporally reliable (test/retest *r* > 0.70) among college samples without obese participants over periods between 2 weeks to 2 years (2). Cronbach’s α for the present study was 0.79 and total scores ranged from 1.00 to 27.00.

#### Weight dissatisfaction

Participants were asked how dissatisfied they were with their weight (1 = *not at all dissatisfied*, 10 = *very dissatisfied*). This single item was adapted from Heinberg and Thompson’s visual analog scale (34).

#### Social comparison tendency

We employed the 11-item Iowa–Netherlands Comparison Orientation Measure (INCOM) to measure social comparison tendencies (35). This scale includes both positively (e.g., “I always pay a lot of attention to how I do things compared with how others do things”) and negatively worded items (e.g., “I am not the type of person who compares often with others”). Participants responded on a five-point scale, response items ranged from (1) *strongly disagree* to (5) *strongly agree*. Negative items were reverse-scored so that a higher total indicates higher comparison tendencies. Gibbons and Buunk reported adequate temporal reliability (1-month test/retest *r* = 0.71) and good internal consistency among college students (Cronbach’s α = 0.80) (35). Cronbach’s α in the present study was 0.79.

#### Body image investment

Participants completed the 20-item Appearance Schema Inventory-Revised (ASI-R) (27). This scale measures the belief that appearance is important, meaningful, and influential. Sample items include “my appearance is responsible for much of what’s happened to me in my life” and “when it comes to my physical appearance, I have high standards.” Participants responded to each item on a five-point scale ranging from (1) *strongly disagree* to (5) *strongly agree*. Negatively worded items were reverse-scored prior to analyses so that a higher score indicates greater investment. Cash and colleagues reported adequate internal consistency for this inventory (Cronbach’s α = 0.88) (27). Cronbach’s α in the current study was 0.86.

#### Eating expectancies

Participants completed four subscales from Hohlstein, Smith, and Atlas’ Eating Expectancy Inventory that measure whether or not eating is used as a reinforcer (31). Two of these subscales assess eating as a negative reinforcer (eating helps manage negative affect and eating alleviates boredom), and two assess eating as a positive reinforcer (eating is pleasurable/a useful reward and eating enhances cognitive competence). Participants responded to each item on a seven-point scale ranging from (1) *strongly disagree* to (7) *strongly agree*. Any negatively worded items (e.g., “eating does not make me feel out of control”) were reverse-scored prior to analyses, meaning that participants with a high score strongly endorsed these expectancies. Hohlstein et al. reported high internal consistency estimates for each of the four subscales (Cronbach’s α range 0.78–0.94) (31). In the current study, Cronbach’s α for the subscales ranged from 0.78 to 0.93.

#### Food intake

After completing the questionnaires online, participants were invited to partake in a *supposedly* independent study on taste perceptions 2 weeks later. Alone in the laboratory, participants completed a 10-min taste test with two large pre-weighed bowls of crispy (i.e., wafer and chocolate) and original chocolate M&Ms. To encourage food intake, participants were required to taste and rate the M&Ms on a variety of different dimensions (e.g., salty, sweet); they were informed that we had plenty of M&Ms and that the two bowls would not be used for another participant’s taste test. The total grams consumed from each bowl were combined to form one food intake variable.

### Analyses

We employed bivariate (Pearson) correlations to test the relationships between restraint scores and the individual variables. After calculating bivariate correlations, Stieger’s *z*-tests for correlated correlations were conducted to investigate whether or not each pair of correlations differed significantly from one another. Three age outliers (>39.71 years) were retained in the analyses because their removal did not affect the statistical significance of the *z*-test results.

## Study 1 Results and Discussion

The DIS and the RS correlated highly with each other (*r* = 0.67). The correlations between restraint scores and the other variables are displayed in Table 1 and we have based our interpretations on the *z*-test results, which compare the correlations. Evidently, both restraint scores correlated with weight dissatisfaction and body-image investment. The *z*-tests demonstrated that such correlations (including those with social comparison) did not differ significantly as a function of which restraint scale scores were used. However, the positive correlations between both restraint scores and BMI differed significantly depending on which restraint scale was used. The correlation with BMI was significantly larger when using their RS scores (*r* = 0.39; DIS *r* = 0.11).

**Table 1:** **Study 1: *Z*-test results and Pearson correlations**.

	Correlations		*Z*-test
	RS	DIS	*Z*
BMI	0.39***	0.11	3.69***
Weight dissatisfaction	0.50***	0.43***	0.92
Social comparison^a^	0.27**	0.16	1.45
Body-image investment^a^	0.42***	0.39***	0.37
EE boredom	0.29**	0.19*	1.31
EE negative affect	0.34***	0.15	2.60**
EE reward	−0.09	−0.24*	2.06*
EE cognitive	−0.12	−0.24*	1.61
Taste test food intake (g)	−0.00	−0.02	0.22

*EE, eating expectancy*.

**Z* = Steiger’s *Z*-test for correlated correlations within a population. *z*-critical values for *p* < 0.05, *p* < 0.01, and *p* < 0.001 are: 1.96, 2.58, and 3.29, respectively*.

*^a^Variable correlated significantly with age (*r* = −0.20, *p* = 0.04), but partial correlations did not affect results and are not presented*.

*p < 0.05.

**p < 0.01.

***p < 0.001.

According to the *z*-test results, there was no significant difference between how the two restraint scores correlated with endorsement of the expectancies that eating relieves boredom and that eating enhances cognitive competence. However, the correlations between participants’ restraint scores and the endorsement of the other two eating expectancies did differ significantly as a function of which restraint score was used. The positive correlations between restraint scores and the expectancy that eating alleviates negative affect were significantly larger when using RS scores (*r* = 0.34; DIS *r* = 0.15). It is noteworthy that this eating expectancy is related to dietary disinhibition (31). Conversely, participants who scored highly on the restraint scales tended to be less inclined to endorse the positively reinforcing expectancy that eating is pleasurable and useful as a reward, an expectancy that is also associated with disinhibition (31). These *negative* correlations were significantly larger when conducted with DIS (*r* = −0.24), compared to RS scores (*r* = −0.09). Perhaps this significant difference suggests that the DIS is more capable of tapping into dietary inhibition, than is the RS.

Neither restraint score correlated significantly with food intake during the taste test and the obtained correlations did not differ significantly as a function of which restraint scale was used. In hindsight, perhaps the one unhealthy option (chocolate) was a limitation in Study 1. Participants may have felt like eating more food, but they were not tempted by the M&Ms on offer. Previous researchers have shown that individuals get bored when they are only offered one type (vs. a variety) of food and that this boredom triggers a statistically significant decrease in consumption (36). Therefore, a lack of food-choice variety during taste tests might mask or inhibit participants’ true eating behavior. This limitation was remedied in the second study.

## Study 2 Introduction

In addition to redesigning the taste test for Study 2, we measured participants’ 18-month weight change. We also reassessed a selection of the above self-report variables. So as not to overburden participants, we kept the number of items that they completed similar to the number of items in Study 1. We removed the measures of body-image investment and eating expectancies from the questionnaire and replaced them with self-report measures of thin-ideal internalization, self-regulation, and self-control. All three of these constructs have been implicated in the dietary restraint literature (37–40) and it is important to directly compare how different restraint scales tap into such individual differences.

## Study 2 Method

### Participants

Although 249 participants completed this study, 7 underweight participants (BMI < 18.5) and 26 participants with a BMI ≥ 30 were excluded from data analyses. The final sample consisted of 216 participants with a mean age of 20.80 (SD = 6.83, range 17–59) and a mean BMI of 23.35 (SD = 2.81, range 18.59–29.51). One hundred and fifty-one participants were classified as normal weight, and 65 as overweight (BMI ≥ 25). Eighty-one percent of the sample identified themselves as New Zealand European, 5% as New Zealand European and New Zealand Māori, 4% as Chinese, and the remaining 10% of participants identified as other ethnicities (e.g., North American). As in Study 1, this sample was not screened or selected for weight-loss or dieting intentions.

### Measures and procedure

This study was approved by the campus Human Ethics Committee. Study 2 recruitment was similar to Study 1’s recruitment. Participants were offered $10 or course credit to complete the study. Data were collected alongside of another study, which was advertised as a personality and sensory awareness study (e.g., sight and taste) (41). Participants completed the following self-report measures online: the DIS, the RS, and measures of weight dissatisfaction, social comparison tendencies, thin-ideal internalization, perceived self-regulatory success, and dispositional self-control. As in Study 1, 2 weeks later, they also completed a taste test as part of a *separate* study and, prior to debriefing, the investigator weighed participants and measured their height to calculate a BMI. The same health-related contact information that was provided to Study 1 participants was also given to Study 2 participants.

Eighteen months after the initial study, participants received an email inviting them to complete a short follow-up questionnaire about their health behaviors. Although this follow-up questionnaire was not limited to “dieting” participants, it is worth mentioning that 23% of those who completed this additional questionnaire self-reported that they were trying to lose weight at the time of follow-up. After participants reported their smoking status (filler item), they self-reported their current weight and then proceeded to complete questions about their exercise habits and fruit/vegetable intake (filler items). One hundred and fourteen participants (53% retention rate) completed this follow-up questionnaire. Returnees and non-returnees did not differ significantly on the measures of age, BMI, RS, weight dissatisfaction, social comparison orientation, self-regulation, or self-control. Returnees recorded significantly lower DIS (2.00) and internalization (24.75) scores than did non-returnees (2.28, 28.41, respectively), *t*s > 2.01, *p*s < 0.01.

#### Dietary restraint status

Participants completed both the DIS (Cronbach’s α = 0.89, range 1.00–4.44) and the RS (Cronbach’s α = 0.81, range 2.00–28.00).

#### Weight dissatisfaction

Participants rated their weight satisfaction on a 10-point scale (10 = *very satisfied*). Scores were reversed prior to analyses so that a higher score indicates greater dissatisfaction.

#### Social comparison tendency

As in Study 1, participants completed the INCOM (35). Cronbach’s α in the current study was 0.74.

#### Thin-ideal internalization

Participants completed the general (not athletic) thin-ideal internalization subscale from the Sociocultural Attitudes Toward Appearance Scale-3 (42). This subscale measures the endorsement of the thin-ideal stereotype projected by the media. Participants rated nine items (e.g., “I wish I looked like the models in music videos”) on a five-point scale (1 = *definitely disagree*, 5 = *definitely agree*). This subscale demonstrates high internal consistency across independent studies (e.g., average α = 0.94) (42). Cronbach’s α in the current sample was 0.92.

#### Dispositional self-control

Participants’ self-control was assessed with a 13-item self-control scale (40). This scale is internally consistent (α = 0.83) and has demonstrated good 3 week test/retest reliability (*r* = 0.87) (40). Cronbach’s α in the current study was 0.81. Items were scored with a five-point scale (1 = *not at all like me*, 5 = *very much like me*) and included positively (“I am good at resisting temptation”) and negatively worded items (“I say inappropriate things”). Positively worded items were reverse scored, meaning that a higher score indicates lower self-control.

#### Perceived self-regulatory success in dieting

This three-item scale was developed by Fishbach, Friedman, and Kruglanski (43). Participants rated the items (e.g., “how successful are you in watching your weight”) on a seven-point scale (1 = *not*, 7 = *very*). Their total score was reversed so that high scores indicate poor self-regulatory success. Cronbach’s α for the present study was 0.66. Although scales with few items have low α-levels (44) and 0.66 is comparable to other research (21), this low Cronbach’s α is a limitation of this measure. Since we conducted the current study, this scale has been re-developed to include “non-applicable” response options for non-weight-concerned participants (38). However, research suggests that participants in past studies likely chose the middle rating (i.e., four) if the item was non-applicable and that the original scale used in the current study is still reliable (38).

#### Food intake

As in Study 1, participants individually completed a 10 min taste test *supposedly* testing the connection between their personalities and taste perceptions – this study was presented as a study independent to the questionnaires (41). Participants were provided with four pre-weighed bowls of high-calorie foods to snack on during the taste test: chocolate/peanut M&Ms, bite-sized cookies, salted pretzel bows, and savory crackers. Because the pieces of food differed in weight, we standardized the grams consumed from each bowl before analyzing the data (45). We formed one combined food intake variable by summing the grams consumed from each bowl of food.

#### Weight change

Eighteen-month body weight was self-reported in the follow-up questionnaire and was used to calculate weight-change (Time 2 weight – Time 1 weight); a positive value indicates weight gain.

### Analyses

As in Study 1, we used bivariate (Pearson) correlations and Stieger’s *z*-tests for correlated correlations. Six age outliers (>41.28 years) and one weight-change outlier (>16.79 kg) were identified in this dataset. However, these data were retained for analyses because their removal did not affect the statistical significance of the *z*-test results.

## Study 2 Results and Discussion

Correlations are presented in Table 2, and our conclusions are based on the *z*-test results comparing correlations. The two restraint scores correlated positively with each other (*r* = 0.66) and the correlations between the restraint scores and scores on the thin-ideal internalization and social comparison scales did not differ significantly as a function of which restraint scale was used. However, the positive correlations between the restraint scores and BMI (RS *r* = 0.33, DIS *r* = 0.20) *and* self-reported weight dissatisfaction (RS *r* = 0.65, DIS *r* = 0.45) were significantly larger when using participants’ RS than when using their DIS scores.

**Table 2:** **Study 2: *Z*-test results and Pearson correlations**.

	Correlations		*Z*-test
	RS	DIS	*Z*
BMI	0.33***	0.20**	2.37*
Weight dissatisfaction	0.65***	0.45***	4.24***
Social comparison^a^	0.31***	0.22***	1.52
Thin-ideal internalization^a^	0.43***	0.36***	1.31
Poor self-control^a^	0.21***	0.03	3.11**
Poor self-regulatory success^a^	0.27***	0.02	4.54***
Taste test food intake (g)	0.12	−0.00	2.18*
Weight change (kg)	−0.12	−0.22*	1.39

**Z* = Steiger’s *Z*-test for correlated correlations within a population. *z*-critical values for *p* < 0.05, *p* < 0.01, and *p* < 0.001 are 1.96, 2.58, and 3.29, respectively*.

*^a^Variable correlated significantly with age (*r* > 0.21, *p* < 0.003), but partial correlations did not affect results and are not presented*.

*p < 0.05.

**p < 0.01.

***p < 0.001.

In addition, the correlations between restraint scores and dispositional self-control and dietary self-regulation differed significantly between the two restraint scales. Similar to previous research (23), participants’ RS scores showed significantly larger correlations with poor self-control (*r* = 0.21) and poor dietary self-regulation (*r* = 0.27) than did DIS scores (*r* = 0.03 and *r* = 0.02, respectively). Although we acknowledge that both the self-control and self-regulation scales were self-report, it is telling that neither restraint scale was associated with higher reports of control. Moreover, the correlations between participants’ restraint scores and objectively measured unhealthy food intake also differed significantly depending on which restraint score was used. The correlation based on the RS (*r* = 0.12) was significantly higher than that based on the DIS (*r* < −0.01). Participants’ 18-month weight change ranged from −16.40 to 19.20 kg (*M* = −0.15, SD = 5.64) and was unrelated to time 1 BMI (*r* = −0.06). There was a statistically significant negative correlation between their DIS scores and weight change (*r* = −0.22), but not between RS and the weight-change variable (*r* = −0.12). However, when the two correlations were directly compared, they were not statistically significantly different from one another. Therefore, both restraint scales predicted a similar amount of weight-change among participants; 23% of whom reported that they were trying to lose weight at the time of follow-up.

## General Discussion

To advance the dietary restraint literature, it is necessary to know if the various restraint scales are conceptually different from one another and what constructs each scale measures. We compared the well-known RS to the lesser known DIS. Consistent with previous research (8, 23), the two restraint scale scores correlated highly with one another. In addition, both restraint scores correlated similarly with the measures of body-image investment, social comparison tendencies, thin-ideal internalization, and with 18-month weight change among a follow-up subsample containing both participants who were and were not trying to lose weight. Therefore, neither restraint scale was associated with weight *gain* as past research has found with the RS in non-clinical samples (15).

The strength of associations between restraint scores and weight dissatisfaction and BMI did differ as a function of restraint scales. Furthermore, the relationship between participant’s restraint scores and the range of variables related to unhealthy (over)eating (e.g., the expectancy that eating alleviates negative affect, poor dispositional self-control) were stronger when they were calculated with participants’ RS, rather than DIS scores, and the correlation between restraint scores and objectively measured food intake was also significantly different as a function of which restraint scale was used (Study 2).

Based on self-reported restraint scales, past researchers have alleged that weight-loss dieting is counterproductive and that it encourages increased food intake and weight gain – i.e., restraint-related effects (6, 20). Among our non-clinical student sample (not selected for weight-loss intention or dieting status), the results suggest that such conclusions cannot be based on data derived from the RS or the DIS. Although it was only the RS, which correlated with poor self-control, neither scale measured successful self-control or dietary regulation (i.e., dieting), though nor did they tap in to *weight gain*. At first glance, our results might suggest that Stice achieved his goal of developing a restraint scale distinct from overeating; however, on closer inspection, it is still unclear what exactly the DIS measures. Aside from the negative correlations between participants’ DIS scores and 18-month weight change and positively reinforcing eating expectancies, the majority of results did not indicate that participants’ DIS scores correlated with typically “successful” variables such as dispositional self-control or limited food intake. That is, even though it is noteworthy that the scale items did not measure dietary disinhibition, neither did they relate to subjective or objective measures of dietary control.

### Strengths, limitations, and implications

Our studies were strengthened by the variety of subjective and objective measures, and by the longitudinal follow-up in Study 2. However, it is important to note that the majority of conclusions were based on self-report data and that results can only be generalized to normal weight and overweight women. In addition, correlational analyses cannot determine cause and effect, and their results often vary with sample size. The correlations between the restraint scales, weight satisfaction and food intake, respectively, differed as a function of which restraint scale was used in Study 2 only. In addition, there was some variation in the strength of correlations between the studies (e.g., between the DIS and BMI). However, additional Fisher *r*- to *z*-transformations (assessing the difference between two correlation coefficients found in two independent samples) revealed that none of the correlations were significantly different between the two studies, all *z*’s < 1.91, *ns*. Future researchers would benefit from replicating our studies with larger samples. Finally, because our interest lay in the “normal” population of women, we purposely recruited a sample unselected for dieting status or eating pathology. It was nevertheless a limitation that neither dieting status nor disordered eating was measured for descriptive purposes. Regardless of these limitations, our results have applied implications for non-clinical samples of normal and overweight women. Between-study comparisons about restrained eaters identified with different restraint scales need reinterpretation. Although this is not a new statement (2) and it was already very clear that high RS scores relate to overeating (20), we have extended this body of literature to encompass the DIS, which is becoming an increasingly popular research tool. It is important to highlight that neither scale measures successful “dieting” as a layperson would consider dieting. Conclusions and recommendations about weight-loss dieting should not be based upon studies employing dietary restraint scales. Instead, such inferences need to be based on studies that use more validated techniques to measure dietary restriction, such as food diaries or doubly labeled water. We cannot recommend either scale to measure dieting behaviors that lead to a negative energy balance and produce weight loss.

## Author Contributions

Under the supervision of RK and DG, JB completed these studies as part of her Ph.D. thesis. JB conducted the literature search, designed the studies, analyzed the data, and wrote the first draft of the manuscript. DG and RK reviewed the data analysis and contributed to the revision of the manuscript. All authors have approved the final manuscript.

## Conflict of Interest Statement

The authors declare that the research was conducted in the absence of any commercial or financial relationships that could be construed as a potential conflict of interest.
